# Microstructural Characterization of Additively Manufactured Metal Components Using Linear and Nonlinear Ultrasonic Techniques

**DOI:** 10.3390/ma15113876

**Published:** 2022-05-29

**Authors:** Seong-Hyun Park, Sungho Choi, Dong-Gi Song, Kyung-Young Jhang

**Affiliations:** 1 Center for 3D Printing Nondestructive Testing, Korea Advanced Institute of Science and Technology, Daejeon 34141, Korea; seonghyun@hanyang.ac.kr; 2 Department of Mechanical Convergence Engineering, Hanyang University, Seoul 04763, Korea; dgsong@hanyang.ac.kr; 3 LANL-JBNU Engineering Institute-Korea, Jeonbuk National University, Jeonju-si 54896, Korea

**Keywords:** microstructural characterization, linear ultrasonic technique, nonlinear ultrasonic technique, additive manufacturing

## Abstract

Metal additive manufacturing (AM) is an innovative manufacturing technology that uses a high-power laser for the layer-by-layer production of metal components. Despite many achievements in the field of AM, few studies have focused on the nondestructive characterization of microstructures, such as grain size and porosity. In this study, various microstructures of additively manufactured metal components were characterized non-destructively using linear/nonlinear ultrasonic techniques. The contributions of this study are as follows: (1) presenting correlation analyses of various microstructures (grain size and texture, lack of fusion, and porosity) and ultrasonic properties (ultrasonic velocity, attenuation, and nonlinearity parameters), (2) development of nondestructive microstructural characterization techniques for additively manufactured components; and (3) exploring the potential for the online monitoring of AM processes owing to the nondestructive nature of the proposed technique. The performance of the proposed technique was validated using additively manufactured samples under varying laser beam speed conditions. The characteristics of the target microstructures characterized using the proposed technique were consistent with the results obtained using destructive optical microscopy and electron back-scattered diffraction methods.

## 1. Introduction

Metal additive manufacturing (AM), also referred to as metal 3D printing, is an emerging technology for producing metal components [1]. AM is used to produce 3D metal components via layer-by-layer deposition of metal wires, sheets, or powders, wherein a target region is melted using a heat source, such as a laser, arc, or electron beam [2]. AM facilitates innovative, flexible, and lightweight designs for 3D structures with minimal tooling, reduced material waste and lead times, and fast prototyping compared with conventional subtractive or casting manufacturing [3].

Despite the many advantages of AM, the quality assurance of AM components is a significant barrier to its adoption [4]. Typically, the presence of inhomogeneous microstructures in the interior of AM components owing to insufficient energy irradiation, material overheating, or prolonged exposure to varying temperatures in the deposited layer, results in uneven grain structures, gas pores, and a lack of fusion (LOF) [5] in the final products. To address these problems, several nondestructive testing (NDT) methods, such as vision-based testing [6], pyrometry [7], thermography [8], and radiography [9] have been proposed. Although the aforementioned NDT methods are effective for evaluating melt-pool characteristics, surface discontinuity, and subsurface cracks in AM components, these features are not highly relevant to the microstructural characteristics.

Ultrasonic NDT is the most powerful method for characterizing the structures of materials [10,11]. A linear ultrasonic technique, which uses ultrasonic velocity or attenuation coefficient measurements to characterize internal defects [12], phase changes [13], cracks [14], porosity [15], and grains [16], has been extensively investigated for conventional subtractive materials. In addition, nonlinear ultrasonic techniques have been investigated to characterize microcracks [17], microporosity [18], and several microstructures, including dislocation density [19], precipitates [20], and grain boundaries [21].

In contrast to when using conventional materials, the thermomechanical manufacturing process of AM causes multiple microstructural properties to evolve concurrently [22]. With respect to AM materials, several case studies have reported the use of ultrasonic NDT. Slotwinski et al. [23] and Karthik et al. [24] correlated the ultrasonic velocity with the material density of the AM samples. Javidrad et al. [25] correlated the ultrasonic velocity and anisotropic elastic constants with the porosity distribution. Foster et al. [26] evaluated the interfacial bonding quality between interlayers based on ultrasonic velocity measurements for ultrasonic AM components. Smith et al. [27] used spatially resolved acoustic spectroscopy to detect the internal defects. However, these studies focused on the evaluation of macroscale defects, such as macroscale pores and voids, and material density degradation. Stratoudaki et al. [28] and Pieris et al. [29] applied a laser-induced phased array system for remote inspection. Park et al. [30] used femtosecond laser ultrasonics with laser polishing for an online mechanical property estimation during the AM process. In addition, Sotelo et al. [31] assessed the hardness differences in wrought, AM, and hybrid AM samples using ultrasonic velocity, attenuation, and backscatter measurements. Bellotti et al. [32,33] evaluated the dislocation densities of wrought and heat-treated AM components using a nonlinear ultrasonic technique. Although these studies successfully analyzed the differences in material properties between wrought and AM components using ultrasonic NDT, no studies have explicitly focused on characterizing the various microstructural features that occur during the AM process.

In this study, various AM metal component microstructures were characterized using ultrasonic NDT. We used two linear ultrasonic properties (ultrasonic velocity v and ultrasonic attenuation coefficient (a) and one nonlinear ultrasonic property (ultrasonic nonlinearity parameter (β) to characterize various microstructural features, which were determined by controlling the AM laser beam speed (LBS). From the correlation analysis, an effective ultrasonic NDT was proposed for microstructural characterization. Owing to its nondestructive nature, the proposed technique demonstrates the potential for online monitoring of AM processes.

The remainder of this paper is organized as follows. Section 2 presents a brief description of ultrasonic NDT methods using ultrasonic velocity (v), attenuation (a), and nonlinearity (β) parameters. Section 3 describes the experimental setup, such as the fabrication of test samples, ultrasonic measurements, and metallography methods. The experimental metallography results, including optical microscopy (OM) and electron back-scattered diffraction (EBSD), ultrasonic signal acquisitions, correlation analysis, and microstructural characterization using ultrasonic NDT, are discussed in Section 4. Section 5 presents the conclusions, limitations, and future research directions.

## 2. Description of Ultrasonic Velocity, Attenuation, and Nonlinearity Parameters

In this study, the three ultrasonic parameters v, a, and β were used to characterize the microstructures, such as the grain size, texture, LOF porosity, and microporosity of the AM components. Parameter v is a typical linear ultrasonic property that describes how the arrival time of the propagating ultrasonic wave in the material varies [34]. It directly correlates to the material density and linear elastic properties of the material that are affected by the porosity content and distribution [35], and the grain size and texture [30] and can be obtained as [36]:

(1) $$v=\frac{x}{t},$$

where x is the ultrasonic propagation distance and t is the arrival time of the ultrasonic wave.

The parameter a, which is another linear ultrasonic property, quantifies the ultrasonic amplitude loss due to scattering, absorption, or friction when ultrasonic waves propagate through a material [16]. a is often dependent on microstructural features such as grain size, boundary [37], textures [38], pores [12], and inclusions [39]. For conventional metals, nondestructive evaluation of various microstructural features using ultrasonic forward or backscattering has been extensively reported [40]. This ultrasonic attenuation has been actively applied by several researchers in AM for melt-pool monitoring during the AM process [41] and mechanical property estimation [30,31]. Generally, the mechanisms of varying a differ according to the ratio of the ultrasonic wavelength and the existing scatterers in the material [10]. Because the range of ultrasonic frequency commonly used for NDT is 2–10 MHz, the ultrasonic wavelengths for common AM metals (stainless steel and titanium alloys) range from 0.6–3 mm, which is larger than the material microstructural features. Then, the Rayleigh scattering theorem becomes a dominant attenuation mechanism and can be obtained as follows [10]:

(2) $$a=-\frac{20}{x}\log\left(\frac{S_{x}{}^{\prime }}{S_{0}{}^{\prime }}\right),$$

where $S_{0}{}^{\prime }$ and $S_{x}{}^{\prime }$ are the initial amplitude of the ultrasonic wave and the amplitude after the propagation distance, respectively.

Parameter β is an index related to the material second-order nonlinear elastic constants [17]. This parameter depends on the monochromatic fundamental ultrasonic wave and the higher harmonic components generated by the distortion of the fundamental wave. Because β is known to be sensitive to the material microstructural characteristics, such as dislocations [19], precipitates [42], microscale cracks [14], and grain boundaries [37], its application has recently been actively investigated. β can be derived as [17]:

(3) $$\beta =\frac{8(A_{2})}{k^{2}x{\left(A_{1}\right)}^{2}},$$

where k is the ultrasonic wavenumber and $A_{1}$ and $A_{2}$ are the displacement amplitudes of the fundamental and second-order harmonic frequency components of the propagating ultrasonic wave, respectively. Owing to the difficulty in precisely measuring ultrasonic displacements, several researchers have used a relative parameter $\beta^{\prime }$ instead of β, which is obtained as follows [43]:

(4) $$\beta^{\prime }=\frac{\left(A_{2}{}^{\prime }\right)}{{\left(A_{1}{}^{\prime }\right)}^{2}}D,$$

where $A_{1}{}^{\prime }$ and $A_{2}{}^{\prime }$ indicate the electrical signal amplitudes for the respective frequency component measured by an ultrasonic detector and D is the ultrasonic attenuation compensation factor. Although $\beta^{\prime }$ is the relative value of ultrasonic nonlinearity, it can be used to compare the before/after microstructural variations under constant material conditions [17]. Notably, ultrasonic attenuation also results in a decrease in $A_{1}{}^{\prime }$ and then results in $\beta^{\prime }$ being overrated during the ultrasonic measurement [44]. To prevent this problem, D is included to distinguish the effects of nonlinear harmonic generation from those of attenuation, which is derived as follows [44]:

(5) $$D=\frac{\left(a_{2}-2a_{1}\right)x}{1-e^{-\left(a_{2}-2a_{1}\right)x}},$$

where $a_{1}$ and $a_{2}$ are the attenuation parameters at the fundamental and second-order harmonic frequencies, respectively. 

## 3. Materials and Methods

### 3.1. Samples

**Figure 1:**
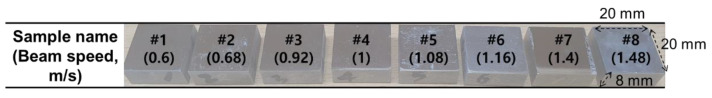
Varying LBS conditions applied to the test samples and their photograph.

Eight test samples with different microstructural characteristics were fabricated using a direct-metal-printing (DMP) 3D-printer (ProX DMP 320; 3D Systems, South Caroline, SC 45, USA) with LaserForm^TM^ 316 L stainless steel powder. The microstructures considered were the grain size, texture, LOF porosity, and microporosity, which are dominantly attributable to the mechanical properties of the AM components [45]. To induce these microstructures in the test samples, we used eight LBS conditions of the 3D-printer: 0.6 m/s, 0.68 m/s, 0.92 m/s, 1 m/s, 1.08 m/s, 1.16 m/s, 1.4 m/s, and 1.48 m/s. They were divided into three groups according to the LBS conditions: low (0.6 m/s and 0.68 m/s), medium (0.92 m/s, 1 m/s, 1.08 m/s, and 1.16 m/s), and high (1.4 m/s and 1.48 m/s) LBS conditions. Each sample was 20 mm long, 20 mm wide, and 8 mm high and labeled as #1 through #8, as shown in Figure 1, according to the LBS conditions used. A previous study reported that variations in the LBS induce various microstructures in the AM components targeted in our study [45]. The other AM process parameters, including the laser power, hatch space, and layer thickness, were fixed at 200 W, 0.1 mm, and 0.03 mm, respectively. Notably, ultrasonic measurement is inconsistent under the rough surface conditions of the test samples because the signal-to-noise ratio is lower in the case of a rough surface [46]. To avoid this problem, the tested samples were treated via wire electrical discharge machining, wherein the arithmetic mean roughness (Ra) of the surfaces was smoothed to approximately 0.33 μm (initial Ra: approximately 5.0 μm).

### 3.2. Ultrasonic Measurements

**Figure 2:**
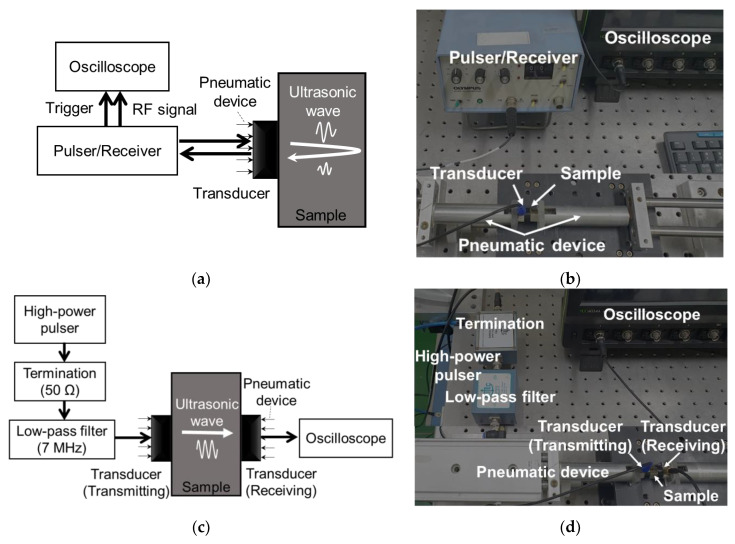
Schematics (**a**,**c**) and photographs (**b**,**d**) for the PE and TT modes, respectively.

In this study, pulse-echo (PE) [47] and through-transmission (TT) [48] modes were used, where the former measures parameters v and a and the latter measures parameter $\beta^{\prime }$. For the PE mode, a single ultrasonic transducer was used, and two consecutive back-wall echoes were measured. Here, the respective x (ultrasonic propagation distance) and t (arrival-time) correspond to twice the sample thickness and time-of-flight (TOF) between two consecutive echoes. Figure 2 shows the experimental setup for the PE and TT modes. A commercial pulser/receiver generated an electrical pulsed signal and excited a commercial piezoelectric contact transducer with a frequency of 5 MHz. A longitudinal ultrasonic wave excited by the transducer was transmitted to the test sample, propagated through it, and reflected on the other side. The ultrasonic signals of the first and second back-wall echoes reflected were received by the same transducer and pulser/receiver and then digitalized on a commercial oscilloscope with a 0.1 ns time resolution and 300 times averaging. The measurements were repeated 10 times for each sample. Because the imperfect contact conditions between the test sample and transducer cause a measurement error owing to the multireflection and attenuation in the contact gap [49], a specially designed pneumatic device was used, in which the contact condition in each measurement was constant at 0.4 MPa, as shown in Figure 2b. Notably, when the ultrasonic wave propagates over a certain distance (the so-called Fresnel near-field zone [43]), the ultrasonic wave in a material may diffract and result in diffraction attenuation, which is independent of the microstructural characteristics. Here, the wave propagation distance is 16 mm (twice the sample thickness) within the Fresnel zone (approximately 19 mm [43]); therefore, this effect was neglected.

The $\beta^{{}^{\prime }}$ parameter was measured using the TT mode, wherein a 5 MHz transducer was used as the transmitter and a 10 MHz transducer was used as the detector to measure the second harmonic component more suitably. In the TT mode, an ultrasonic wave with a long pulse length is used to obtain a narrower frequency spectrum than that of the PE mode because the narrow-band spectrum is beneficial for dividing the first and second harmonic components in the frequency domain [43]. Therefore, a voltage sinusoidal signal comprising nine cycles was used in the TT mode, generated by a pulser (RAM-5000; RITEC, Warwick, 44-74300, UK) comprising a synthesizer and high-power gated amplifier. This signal passed through a 12 dB attenuator, a high-power 50 Ω termination, and a 7 MHz low-pass filter to suppress the initial harmonic frequency components generated in the pulser and drive the 5 MHz transmitting transducer. Suppressing the initial harmonic components is important for measuring just the harmonic components that correspond to the microstructural characteristics of the test samples [17]. The wave propagated through the sample was received on the other side of the sample by the detector (10 MHz transducer). The measurement was repeated by increasing the input power to obtain a linear fitting plot of ${\left(A_{1}^{\prime }\right)}^{2}$ and $A_{2}{}^{\prime }$ (proportional to $\beta^{\prime }$). A specially designed pneumatic control system was also used to obtain accurate measurements [50]. Here, the D value (attenuation compensation factor (*D*)) was obtained from the aforementioned PE mode. A schematic and a photograph of the TT mode are shown in Figure 2c,d.

### 3.3. Metallography

After the ultrasonic measurements, X-ray diffraction (XRD), OM, and EBSD were performed on small samples, which were detached from the test samples for a comparison with the ultrasonic results. Phase identification was performed using a commercial X-ray diffractometer with a step size of 0.01° and 2θ ranging from 20 to 80°. Porosity, grain size, and texture measurements were performed using a commercial optical microscope and EBSD instrument, respectively, at the cross-section parallel to the building direction of the additional samples. The grain sizes and porosity contents were measured and averaged using “Quatro S” and “ImageJ”, respectively, which are commercial software programs for the size measurement. In these procedures, additional mechanical polishing with diamond suspensions was performed on surfaces, down to 0.04 μm.

## 4. Results

### 4.1. Metallography Results

**Figure 3:**
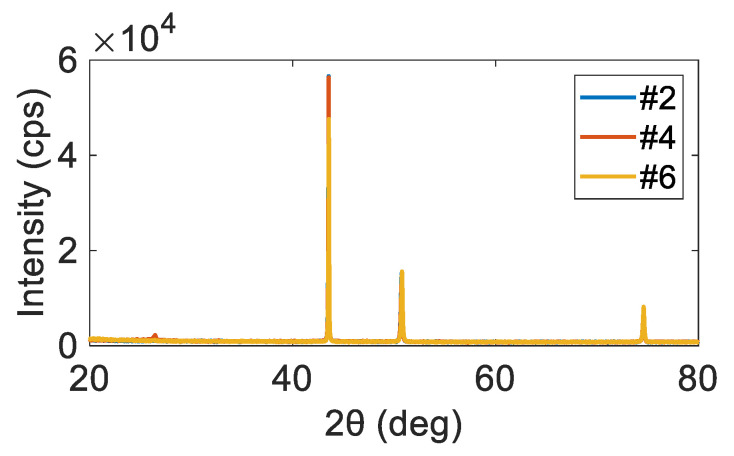
XRD results for the #2, #4, and #6 samples. The major phases with the highest peak intensities of the samples were presented as cubic FCC-austenite γ.

**Figure 4:**
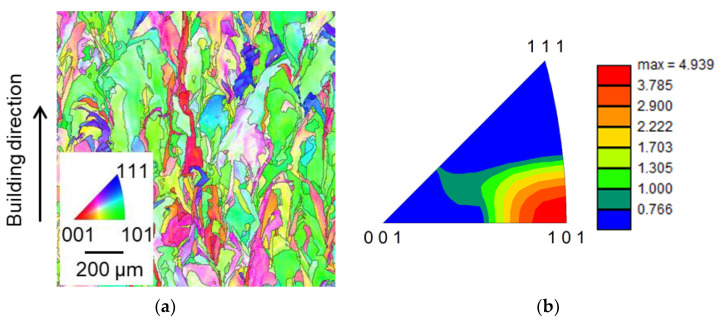

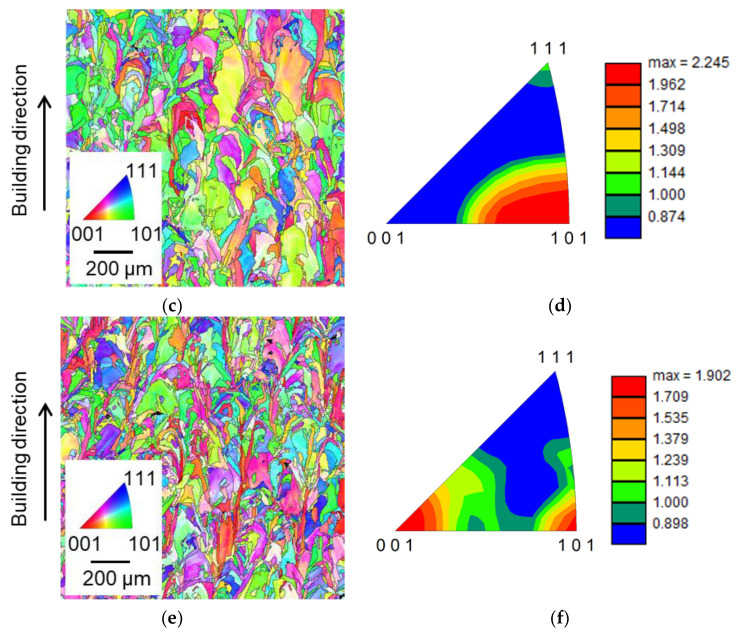
IPF images and corresponding crystallographic textures obtained using EBSD for the 0.6 m/s (**a**,**b**), 1.0 m/s (**c**,**d**), and 1.4 m/s (**e**,**f**) LBS conditions.

An XRD result for the 1.16 m/s LBS condition is presented in Figure 3, which indicates that the major phase with the highest peak intensity of the sample was cubic FCC-austenite γ, in accordance with previous studies [51]. For the other specimens, similar XRD results were observed. Based on the XRD results, the grain size and texture were measured for all the test samples via EBSD. The inverse pole figure (IPF) maps for the typical 0.6 m/s, 1.0 m/s, and 1.4 m/s are presented in Figure 4a,c and Figure 4e, respectively. A large grain size of 105 μm was observed at a 0.6 m/s LBS, reducing to 74 μm and 54 μm with an increase in the LBS. Figure 4b,d,f show the crystallographic textures for the representative 0.6 m/s, 1.0, and 1.4 m/s LBS conditions. A high texture intensity of up to 4.9, along the 101 direction, was observed at a 0.6 m/s LBS, and the textures were more random with the increased LBS conditions. An increase in LBS has been reported to reduce grain size and texture intensity in a specific direction due to the decreased laser energy input in the melt pool and interaction time between the powders and laser, consequently restricting grain growth [52].

**Figure 5:**
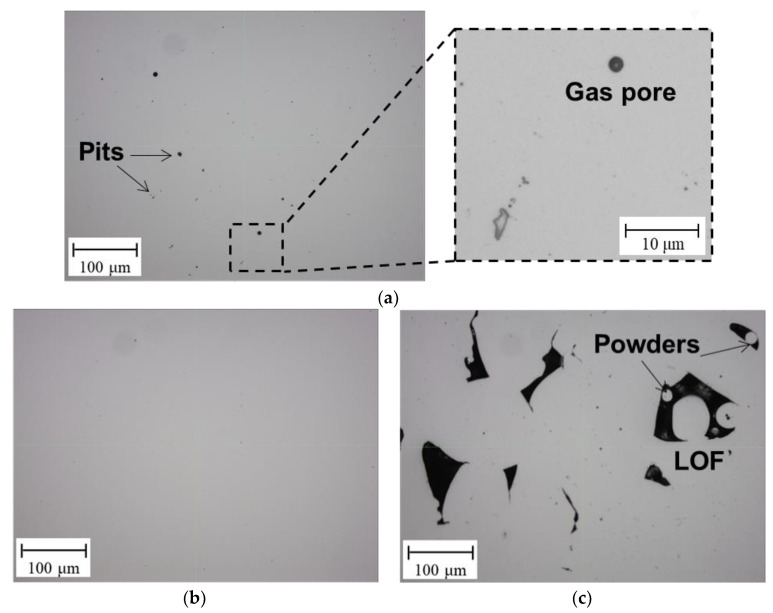
OM images obtained using an optical microscope for the (**a**) 0.6 m/s, (**b**) 1.0 m/s, and (**c**) 1.4 m/s LBS conditions.

The porosity was evaluated using OM and its content was calculated from 2D OM images as the ratio of the pore area to the total area [53]. The typical OM images for the 0.6 m/s, 1.0 m/s, and 1.4 m/s conditions are presented in Figure 5a,b and Figure 5c, respectively. The occurrence of various types of pores, including gas pores and small pits (0.6 m/s LBS), and LOF with unmelted powders (1.4 m/s LBS), indicated by black spots, were observed, whereas almost no pores were observed in the case of the 1.0 m/s LBS. The decreased LBS at 0.6 m/s results in excessive laser energy input in the melt pool, generating various discontinuities, such as wavy surfaces and welded particles on the deposited layer [54]. In general, these discontinuities can be eliminated when the next AM layer is deposited in the following layer (the so-called re-melting process). However, the lower laser-energy input according to the decreased LBS often results in a melt pool with a shallow shape, thus resulting in insufficient penetration of the previously deposited layer [55]. Consequently, pores with small pits were observed between the interlayers. Furthermore, excessive energy input increases gas solubility, resulting in increased gas porosity [56]. The observation of higher porosity contents for the 1.4 m/s LBS condition is attributed to the lack of laser energy input in the melt pool during the AM [54], wherein the insufficient energy causes LOF with the unfused powders between layers or within layers.

The microstructural characteristics of each LBS condition, based on the metallographic results, are summarized in Table 1. Under LBS conditions lower than 0.68 m/s (low LBS condition), approximately 100 μm grain size, approximately 5.2 texture intensity along the 101 direction, and submicrometer microporosity (porosity content: 1.8%) were observed. Under LBS conditions between 0.92 m/s and 1.16 m/s (medium LBS condition), the average grain size and texture intensity along the 101 direction were 68 μm and 2.6 μm, respectively, and almost no pores were observed (porosity content: 1.1%). As the LBS further increased (high LBS condition), the average grain size (53 μm) and texture intensity (1.9 along the 101 direction) decreased, i.e., textures were more random, and submillimeter-scale LOF porosity (porosity content: 7.0%) occurred. Accordingly, each microstructural characteristic was labeled as “State I,” “State II,” and “State III,” respectively, as denoted in Table 1.

### 4.2. Ultrasonic Signal Acquisition Results

**Figure 6:**
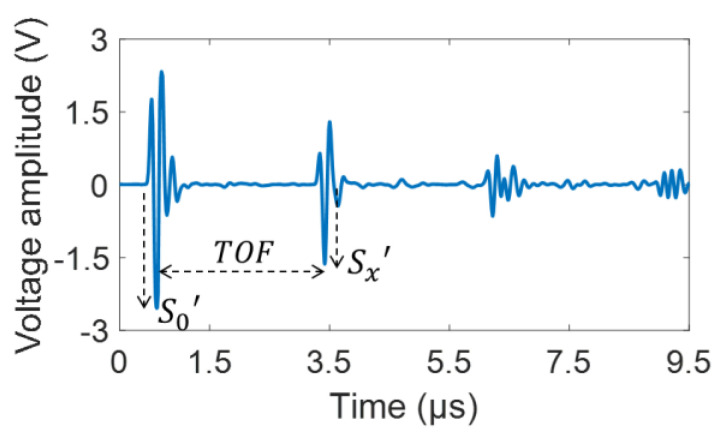
Typical time-domain ultrasonic signal measured from the PE mode.

**Figure 7:**
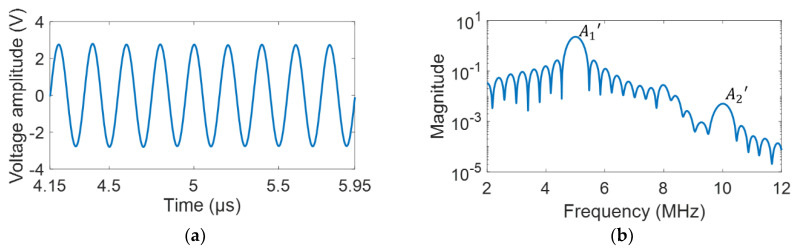

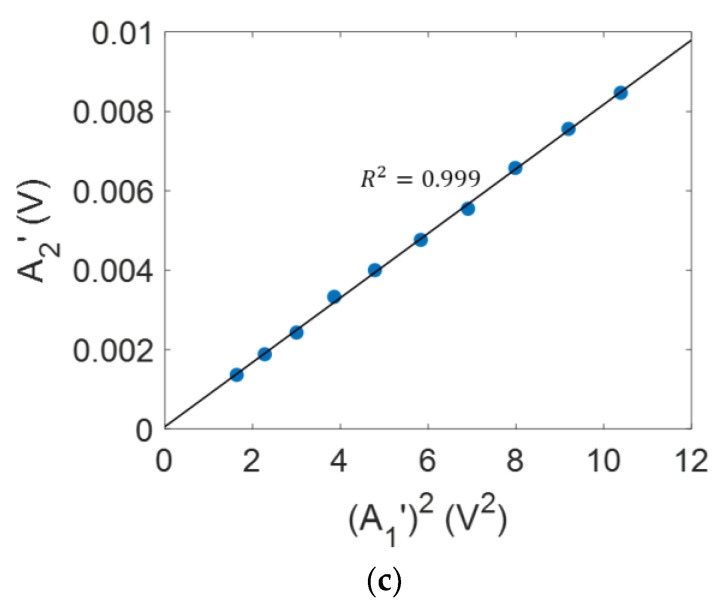
(**a**) Typical time-domain ultrasonic signal measured from the TT mode, (**b**) its frequency spectrum transformed using FFT, and (**c**) relationship between ${\left(A_{1}^{\prime }\right)}^{2}$ and $A_{2}{}^{\prime }$ with the increasing voltage input to obtain parameter $\beta^{\prime }$.

Figure 6 presents an ultrasonic signal acquired from the PE mode, where the parameters v and a were obtained using Equations (1) and (2), respectively. Here, v was calculated from x (ultrasonic propagation distance) and TOF between the first and second peaks of the back-wall echoes, as shown in Figure 6, wherein TOF was measured using a cross-correlation function [24]. When calculating a, the peak amplitudes of the first and second back-wall echoes were used as the $S_{0}{}^{\prime }$ and $S_{x}{}^{\prime }$ values, as shown in Figure 6. Figure 7a presents the ultrasonic signal comprising a sinusoidal wave with nine cycles in the time domain measured from the TT mode. Figure 7b presents the signal in the frequency domain that was Fourier transformed from the time-domain signal using the fast Fourier transform (FFT). Here, a Hanning window was used to obtain a good frequency resolution, thus resulting in high amplitude accuracy in the frequency domain [49]. In the frequency domain, the voltage signal amplitudes for the 5 MHz fundamental and 10 MHz second-harmonic frequency components were extracted and presented as ${A_{1}}^{\prime }$ and ${A_{2}}^{\prime }$, respectively, as shown in Figure 7b. Figure 7c presents the relationship between ${\left(A_{1}^{\prime }\right)}^{2}$ and $A_{2}{}^{\prime }$ with increasing voltage input to extract the $\beta^{\prime }$ parameter. A linear relationship with a high correlation coefficient (0.999) was observed. Finally, $\beta^{\prime }$ was calculated using Equation (4). As mentioned above, the value of D was obtained using the PE mode. Here, $a_{1}$ and a are identical, and $a_{2}$ is obtained using an additional 10 MHz transducer [44].

### 4.3. Microstructural Characterization Using Ultrasonic NDT

**Figure 8:**
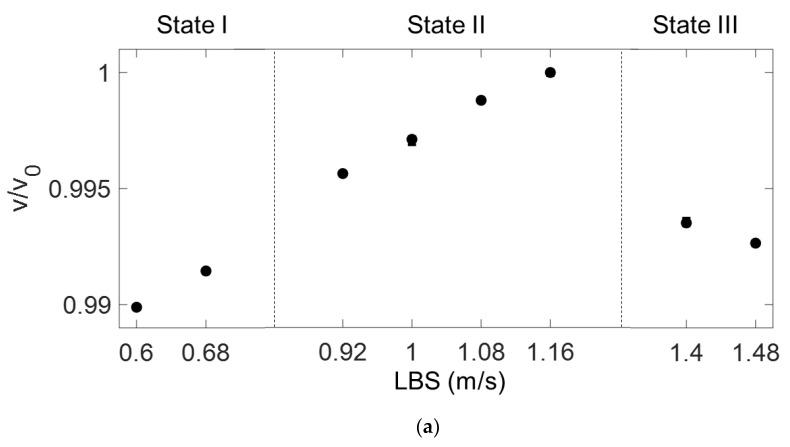

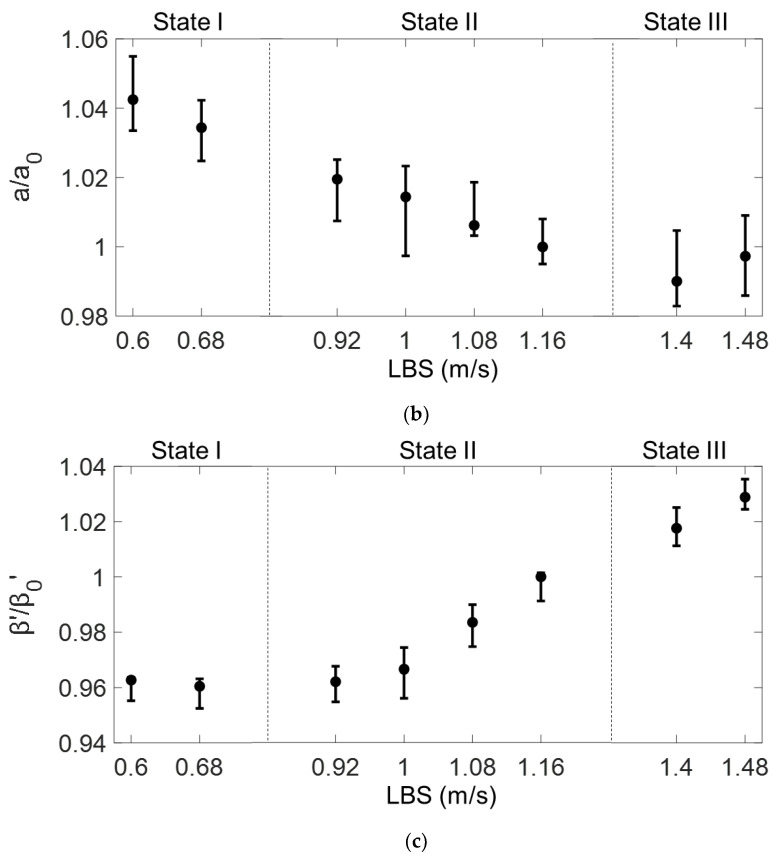
(**a**) Normalized v, (**b**) a, and (**c**) $\beta^{\prime }$ parameters measured according to LBS for each State.

Figure 8a–c presents the parameters v, a, and $\beta^{\prime }$ obtained using the ultrasonic measurements, respectively, according to the varying LBS conditions for State I, II, and III. The *y*-axis values in Figure 8 are the normalized values, which were divided by parameters $v_{0}$, $a_{0}$, and $\beta_{0}^{\prime }$, respectively, where the subscript 0 indicates the measurement at 1.16 m/s LBS.

In Figure 8a, the parameter v increases as the LBS increases from 0.6 to 1.16 m/s (i.e., in State I and II), before sharply decreasing as LBS increases further (i.e., in State III). Although v increased by 0.42% in State II, it sharply decreased by 0.65% in the transition zone of State II and III. At the highest LBS condition of 1.48 m/s in State III, the normalized parameter v was the lowest, i.e., 0.74% less than that at the 1.16 m/s LBS (=$v_{0}$ condition). When State I transitions to State II, almost no slope variation is observed. In Figure 8b, a decreases from State I to the first LBS condition of State III, corresponding to an LBS of 0.6–1.4 m/s; however, a reversed trend is observed as the LBS further increases to 1.48 m/s. In Figure 8c, the $\beta^{\prime }$ value increases as the LBS value increases from 0.92 m/s to 1.48 m/s (in State II and III); however, a notable slope variation is observed at the LBS conditions of 0.6 m/s and 0.68 m/s (in State I) as compared to those of State II and III. Although a 3.8% change in the normalized $\beta^{\prime }$ was observed in State II, only a 0.16% change occurred in the transition zone of State I and II. At the minimum LBS of 0.6 m/s, the slope was reversed, and the normalized $\beta^{\prime }$ increased by 0.23% compared to that for 0.68 m/s LBS. Almost no slope variation was observed between states II and III.

**Figure 9:**
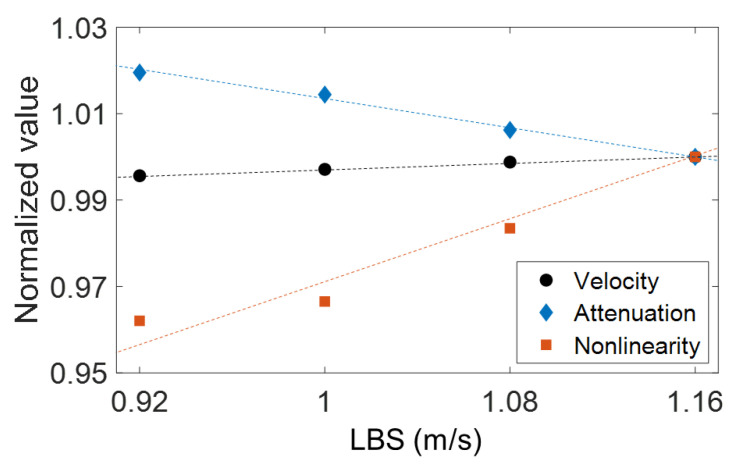
Comparison of the three parameters (v, a, and $\beta^{\prime }$) measured under the LBS conditions between 0.92 m/s and 1.16 m/s (State II).

From the ultrasonic measurement results, ultrasonic parameters that are effective in characterizing the microstructural evolution at the respective states I, II, and III were selected. State II (0.92–1.16 m/s LBSs), where only the grain size and crystallographic texture vary with LBS, was analyzed first. Figure 9 presents a comparison of the normalized v, a, and $\beta^{\prime }$ parameters correlated with varying LBS in State II. The three parameters show a consistent variation, wherein each normalized v, a, and $\beta^{\prime }$ parameter decreased, slightly increased, and increased, respectively. Comparing the average variations for each measured parameter, the most sensitive parameter depending on LBS (i.e., grain size and crystallographic texture) in State II was $\beta^{\prime }$. The absolute variation in $\beta^{\prime }$ with the 0.24 m/s LBS increment was 3.8%, which is approximately nine and two times higher than those of v (0.42%) and a (2.0%), respectively. Because only the grain size and texture varied in State II, it can be concluded that $\beta^{\prime }$ is the most effective parameter for evaluating grain characteristics. Concurrently, despite being less than $\beta^{\prime }$, a also exhibited a good correlation.

**Figure 10:**
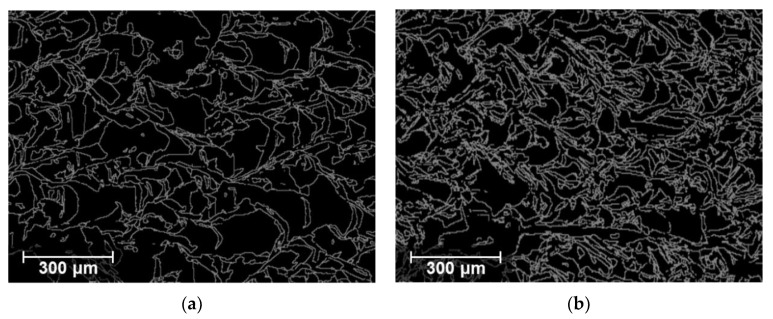
Grain-boundary maps obtained using EBSD for the (**a**) 0.92 m/s and (**b**) 1.16 m/s LBS conditions.

The high sensitivity of $\beta^{\prime }$ in State II (0.92 m/s–1.16 m/s LBSs) is attributed to the relationship between the AM microstructural features, including grain size and texture, and the produced ultrasonic nonlinearity. An increase in LBS leads to a fast cooling rate during the AM process, which results in randomly distributed textures, as shown in Figure 4. These randomly distributed textures have been reported to produce high ultrasonic nonlinearity [33]. Furthermore, these random textures owing to the fast cooling rate have been reported to contain a high dislocation density, which can also contribute to the increased $\beta^{\prime }$ [33]. In addition, a previous study [37] reported that a decrease in the grain size increases the number of grain boundaries in a unit volume, in which the grain boundaries often act as interfacial discontinuities and generate higher harmonics for the propagating ultrasonic wave. To further substantiate this observation, the high-angle grain-boundary maps for the 0.92 m/s (grain size: 75 μm) and 1.16 m/s (grain size: 59 μm) LBS conditions were obtained using EBSD and compared, as shown in Figure 10, where grain boundaries with a misorientation angle exceeding 15° are illustrated by gray lines [57]. More grain boundaries were observed at 1.16 m/s LBS than 0.92 m/s LBS. Although linear ultrasonic properties based on the scattering attenuation mechanism are reportedly sensitive to grain effects in conventional metals [40], $\beta^{\prime }$ was shown to be the most sensitive factor in State II in our experimental results. This may be attributed to the unique thermomechanical manufacturing process of AM, which causes multiple microstructural variations, such as varying grain size and randomly distributed textures; however, further studies are needed to elucidate this observation in detail. A similar observation in AM samples has been reported in the literature [33], which also includes the observation of varying $\beta^{\prime }$ in the AM samples according to the different heat-treatment times. However, the high correlation between the grain characteristics and $\beta^{\prime }$ in the AM samples fabricated under various processing conditions was uniquely observed and investigated in this study.

The second-highest correlation of $a^{\prime }$ in State II is shown in Figure 9. This observation is attributed to the ultrasonic attenuation at the grain boundaries, which has been extensively investigated [10,40]. An ultrasonic wave propagating through the grain boundaries scatters and its amplitude decreases according to the Rayleigh scattering theory, as follows [10]:

(6) $$a=a_{i}+kd^{3}f^{b}$$

where d is the grain size, f is the ultrasonic frequency, and superscript *b* is a constant value determined by the Rayleigh–stochastic zone transition. This theory can explain why a decreased with reducing grain size. This result indicates that the a measurement is a good candidate for replacing the $\beta^{\prime }$ measurement for characterizing State II because of its measurement simplicity [58]. The decreased grain size has been reported to result in a slight increase in v owing to multiple scatterings and the mode conversion of the ultrasonic wave propagating through the grain boundary in a material [59]. Therefore, a correlation between the grain size and v in State II was also demonstrated; however, its sensitivity was less than that of the other parameters.

**Figure 11:**
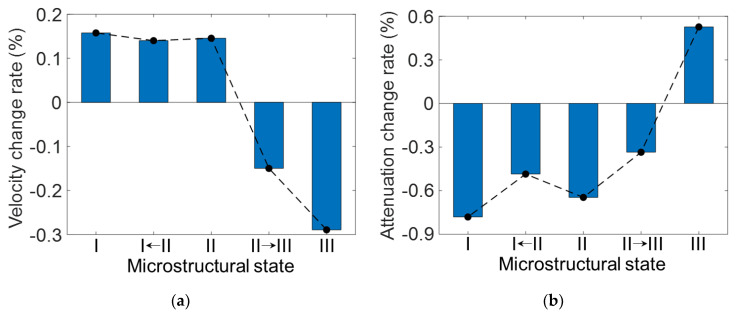

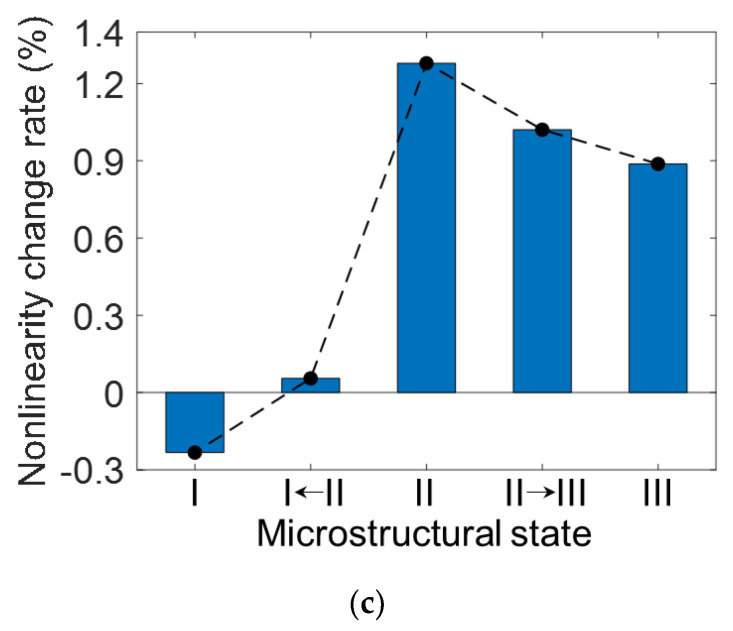
Change rates for the (**a**) v, (**b**) a, and (**c**) $\beta^{\prime }$ parameters measured when State II transitions to State I and III.

When the LBS conditions deviated from State II, the combined effects of the grain size, texture, and porosity on the ultrasonic parameters were observed. Figure 11 presents the trend variations for each measured ultrasonic parameter as State II transitions to State I or III. Here, a label along the *y*-axis represents the change rate of the respective v, a, and $\beta^{\prime }$ parameters, measured according to the 0.08 m/s LBS increment.

When State II transitions to State I (below 0.68 m/s LBS), the grain size and texture intensity along the 101 direction increased, and microporosity occurred. Here, the change rate of $\beta^{\prime }$ according to the 0.08 m/s LBS was the most prominent among the three parameters, as shown in Figure 11c. The change rate of the $\beta^{\prime }$ increment decreased by approximately 1.2% and 1.5% at the transition of State I to II and in State I, respectively, as compared to that of State II. This observation may be attributed to the over-melting porosity generation, the size of which is at the sub-micrometer level, as shown in Figure 5a. The material nonlinear elastic behaviors caused by the imperfect contact between several grains near the micropores can generate higher harmonics of the propagating ultrasonic wave [18]. Furthermore, the closed interfaces of the micropores also generate additional higher harmonics [60]. This type of acoustic nonlinearity is not related to material nonlinearity, such as the grain boundary effect, and results in a much stronger nonlinearity than material nonlinearity. Notably, although the porosity size is very small (at approximately the micrometer level), it can degrade the mechanical properties of the components [5]. In the v and a parameter results, no significant trend variations are observed between State I and II because these parameters are less sensitive than $\beta^{\prime }$ to microvariations in material of several micrometers; thus, these parameters have a stronger relation to grain size variations [58]. If laser energy input greater than that for State I is used during the AM process, the fabricated AM components may be overheated [55]. Under these conditions, not only microporosity but also small pits and bubbles corresponding to the LOF porosity in size (approximately above 100 μm) can be produced. Then, linear ultrasonic properties, including v or a rather than $\beta^{\prime }$, can be effective in damage diagnosis.

In State III (above 1.4 m/s LBS), the grain size and texture intensity along the 101 direction decreased compared to that in State II, and macroscale LOF porosity occurred. Therefore, the ultrasonic parameters were affected to a greater extent by the LOF porosity than by the grain characteristics. In Figure 11a, a drastic decrease in the rate of change of v is observed, i.e., approximately 0.30% and 0.43% at the transition of State II to III and at State III, respectively, as compared to that in State II. This observation is attributed to the LOF porosity, which is in the approximate range of 100 to 200 μm and is much larger than the over-melting porosity (i.e., micropores), as shown in Figure 5c. A large number of LOF pores cause the air-filled cavities inside a component lower the elastic modulus of the material and result in a decreased v according to the following relationship:

(7) $$v=\sqrt{E_{i}\left(1-hV_{p}\right)/\rho },$$

where $E_{i}$ is the elastic modulus obtained from the optimally manufactured component (i.e., porosity-free component), h is a constant value, $V_{p}$ is the porosity content, and ρ is the material density. This relationship indicates that the reduced v results obtained in our experiments are reasonable. In addition, a previous study has also reported that the macroscale porosity is related to an increase in a, which agrees with the observed increase in the increased a result (0.52% increase in the change rate) in State III, as shown in Figure 11b. This is attributed to the additional scattering at the boundary of the LOF porosity, the principle of which is similar to grain scattering (Equation (6)). The relationships between the linear ultrasonic properties, including v and a, and macroscale porosity have been reported in the literature [12]. Owing to the combined effects of the LOF porosity and grain size, no significant increase in a in the transition from State II to III may be observed.

Notably, although $\beta^{\prime }$ is the best indicator in State II, monitoring only $\beta^{\prime }$ may result in a false diagnosis of LOF porosity occurring in the transition zone between State II and III. This is because no notable trend fluctuation for $\beta^{\prime }$ was observed in this transition zone, as shown in Figure 11c. This issue may be addressed via the simultaneous monitoring of $\beta^{\prime }$ and v, which is the best indicator for this transition zone, as mentioned above.

In addition to the microstructural features mentioned above, the different precipitate and residual stress conditions for the tested samples can affect the varying ultrasonic parameters. However, in our experiments, no precipitates were observed in the 316 L stainless steel samples. Generally, precipitates in 316 L stainless steel can be generated in the form of (1) chromium carbides owing to the sensitization heat treatment or (2) delta ferrite under certain manufacturing conditions [22]. However, the samples used did not meet either of the above conditions. Previous studies [61,62] have reported that the residual stress during the AM process primarily depends on different 3D-printing strategies or varying building heights. In our experiments, the above factors were applied equally to all tested samples. Varying AM processing parameters such as laser power and LBS have been reported to contribute to the variation in residual stress conditions [63], which may cause certain experimental errors. To analyze the correlation among AM processing parameters, residual stress, and ultrasonic properties in detail, more detailed experimental designs are required in future studies. Note that when the LBSs are outside the conditions used in this study (from 0.6 m/s to 1.48 m/s), wavy surfaces and large pits were formed in the specimens where ultrasounds could not be detected well.

## 5. Conclusions

In this study, the microstructural characterization of additively manufactured 316 L stainless steel components was investigated using linear and nonlinear ultrasonic techniques. The contributions of this study are the presentation of (1) correlation analyses between various microstructural features (grain size and texture, LOF porosity, and microporosity) and linear/nonlinear ultrasonic properties (ultrasonic velocity, attenuation, and nonlinearity parameters), (2) the development of NDT for the microstructural characterization of additively manufactured components, and (3) the potential for the online monitoring of AM processes owing to its nondestructive nature. The key research outcomes of this study are as follows in Table 2.

The various microstructural features with varying LBSs were characterized using three ultrasonic parameters: v, a, and $\beta^{\prime }$. State I (low LBS condition: below 0.68 m/s) could be effectively characterized using $\beta^{\prime }$ because of the higher harmonics generated owing to the microporosity at levels of several micrometers. The $\beta^{\prime }$ parameter was also effective in characterizing State II (medium LBS condition: 0.92–1.16 m/s) because of the variations in texture, grain boundary, and dislocation density effects. The ultrasonic scattering attenuation at the grain boundary also allowed parameter a to be used for characterizing State II. The generation of the macroscale LOF porosity (approximately greater than 100 μm) in State III (high LBS condition: above 1.4 m/s) showed a good correlation with the v parameter, which was directly related to the material elastic modulus and density.

Further research is required to develop a novel method for the simultaneous monitoring of $\beta^{\prime }$ and v to estimate various microstructural features and use laser ultrasonics for microstructural characterization, which can be conducted in a contactless and nondestructive manner. In addition to the LBS conditions, microstructural variations caused by other process parameters including laser power and hatch space will also be considered in future studies.

## Figures and Tables

**Table 1:** Microstructures according to the varying LBS conditions characterized using metallography.

LBS Condition (m/s)	Low Condition (0.6–0.68)	Medium Condition (0.92–1.16)	High Condition (1.4–1.48)
Microstructural state	State I	State II	State III
Average grain size (μm)	100	68	53
Average texture intensity (101)	5.2	2.5	1.9
Average porosity content (%)	1.8	1.1	7.0
Porosity size	Submicrometers	Almost no pores	Submillimeters
Precipitate	X	X	X

**Table 2:** Summary of effective ultrasonic parameters to characterize each state.

Microstructural State	State I (Microporosity)	State II (Grain Size and Texture Variations)	State III (Macroporosity)
Effective ultrasonic parameters	$\beta^{\prime }$	$\beta^{\prime }$, a	v

## Data Availability

The data presented in this study are available on request from the corresponding author.
